# Impact of age on the circadian visual system and the sleep-wake cycle in *mus musculus*

**DOI:** 10.1038/s41514-021-00063-w

**Published:** 2021-05-04

**Authors:** Dorela D. Shuboni-Mulligan, Demarrius L. Young, Julianie De La Cruz Minyety, Elizabeth Vera, Jeeva Munasinghe, Andrew J. Gall, Mark R. Gilbert, Terri S. Armstrong, DeeDee K. Smart

**Affiliations:** 1 Neuro-Oncology Branch, National Cancer Institute, National Institutes of Health, Bethesda, MD USA; 2 Mouse Imaging Facility, National Institute of Neurological Disorder and Stroke, NIH, Bethesda, MD USA; 3 Department of Psychology and Neuroscience Program, Hope College, Holland, MI USA; 4 Radiation Oncology Branch, Center for Cancer Research, National Cancer Institute, National Institutes of Health, Bethesda, MD USA

**Keywords:** Neural ageing, Ageing

## Abstract

Age plays a critical role in disease development and tolerance to cancer treatment, often leading to an increased risk of developing negative symptoms including sleep disturbances. Circadian rhythms and sleep become disrupted as organisms age. In this study, we explored the behavioral alterations in sleep, circadian rhythms, and masking using a novel video system and interrogate the long-term impact of age-based changes in the non-image forming visual pathway on brain anatomy. We demonstrated the feasibility and utility of the novel system and establish that older mice have disruptions in sleep, circadian rhythms, and masking behaviors that were associated with major negative volume alterations in the non-imaging forming visual system, critical for the induction and rhythmic expression of sleep. These results provide important insights into a mechanism, showing brain atrophy is linked to age in distinct non-image forming visual regions, which may predispose older individuals to developing circadian and sleep dysfunction when further challenged by disease or treatment.

## Introduction

Age is important in disease development and progression^1,2^, as well as response and tolerance to oncologic treatment^3^. Aged individuals are predisposed to develop negative behavioral outcomes, such as disrupted sleep, when exposed to stressors^4,5^. With age, there are progressive and distinct alterations in the expression of sleep patterns with negative effects seen within the homeostatic and circadian systems, which drive sleep behavior^6,7^. Older humans sleep progressively less as they age^8^. This decrease in total sleep time is mirrored in mouse models^9,10^ and accompanied by fragmentation and poorer sleep quality^11^. Such sleep disruptions are associated with increased mortality in older humans^12^. In the circadian system, older individuals also have dampened circadian rhythms^6,13^, alterations in rhythm onset^14^, and increased phase shifting speed^15,16^ but with delayed peripheral clock entrainment^17^. In aged mice, the added stress of jetlag has also been demonstrated to be associated with increased mortality^18^, suggesting the importance of circadian rhythms on the health of older organisms. Understanding the effects of aging on the circadian system is, therefore, important in elderly patients exposed to stressors like oncologic treatments.

The circadian system is comprised of three components^19^, the input entrainment mechanism, the central pacemaker (suprachiasmatic nucleus, SCN), and downstream oscillators within the brain and periphery that feedback to regulate the central pacemaker, all of which are impacted by aging^20^. Aging causes a decrease in the strength of clock gene expression within the SCN^21^ with dampened rhythms in glucose utilization^22^ and in electrical firing rate activity^23,24^. These alterations within the SCN lead to downstream dampening of rhythms within the periphery^17,25^, and the clock-controlled genes which are regulated in a tissue-specific manner. The input mechanism that entrains the SCN in mammals conveys light information solely through the retinal hypothalamic tract (RHT) from the eye^26^. Light signal in the retina destined for the SCN is transmitted through melanopsin containing retinal ganglion cells^27–29^ called intrinsically photosensitive retinal ganglion cells (ipRGCs). Despite the well-established impact of aging on sleep and circadian rhythms, few studies have examined the impact of age on the input system^30,31^, and none have examined the long-term downstream effects within non-image forming regions important for circadian rhythms and masking. Additionally, because many of the studies outlined in our review examining the behavioral analysis of sleep and circadian rhythms are from 20+ years ago, the implementation of updated software to quantify and summarize these behaviors in modern literature is needed.

Recognizing the importance of age in the biology of circadian rhythms, the complexity of the input system, and its downstream effects and associated behavioral manifestations, we explored the relationship between long-term structural changes in the non-image forming visual system using magnetic resonance imaging and age-related alteration in the expression of behavioral circadian rhythms and sleep using a novel system. We hypothesized that deterioration of these structures with age is important in driving deficits observed in behavior, and therefore, could play a role in promoting the development of treatment-related symptoms in older individuals. Here, we validate age-based differences in circadian rhythms, sleep patterns, and masking behavior using a novel video tracking system. Further, we then demonstrated that age impacts the number of retinal ipRGCs which leads to alterations in the brain volume of non-image forming visual regions. These findings suggest that the input system, and specifically the ipRGCs, play a crucial role in the alterations of circadian rhythms, sleep, and masking caused by physiologic aging processes within animals. This novel finding is critical as testing the sensitivity of these regions could aid in the development of strategies to lower the incidence of treatment-related symptoms.

## Results

### Novel video monitoring system for age-based behavioral analysis

Video analysis is a common tool for quantifying behaviors in rodent experiments, however, the use in sleep and circadian rhythms research requires experienced scorers and large quantities of time. Video recording cages with automated behavioral scoring provide instant computer-generated analysis of sleep and activity in real time. The software also provides data for general activity, daily rhythms, sleep, and masking to night-time light exposure. To validate the accuracy of the analysis, the quantification of sleep was compared between manual and automated scoring at the minute level (Supplemental Fig. 2). Both young and old animals had significant correlation between manual and automated scoring. Young animals had significant regression equation (*F*(1, 4781)= 77,114.89, *p* < 0.001), with an *R*^2^ of 0.9328. The residuals in the younger animals were skewed toward predicting more sleep in the automated setting. Old animals had significant regression equation (*F*(1, 5706)= 42,737.06, *p* < 0.001), with an *R*^2^ of 0.882. Older animals had less skewed residuals but had a lower prediction accuracy (−5.08%).

### General activity evaluation

**Fig. 1:**
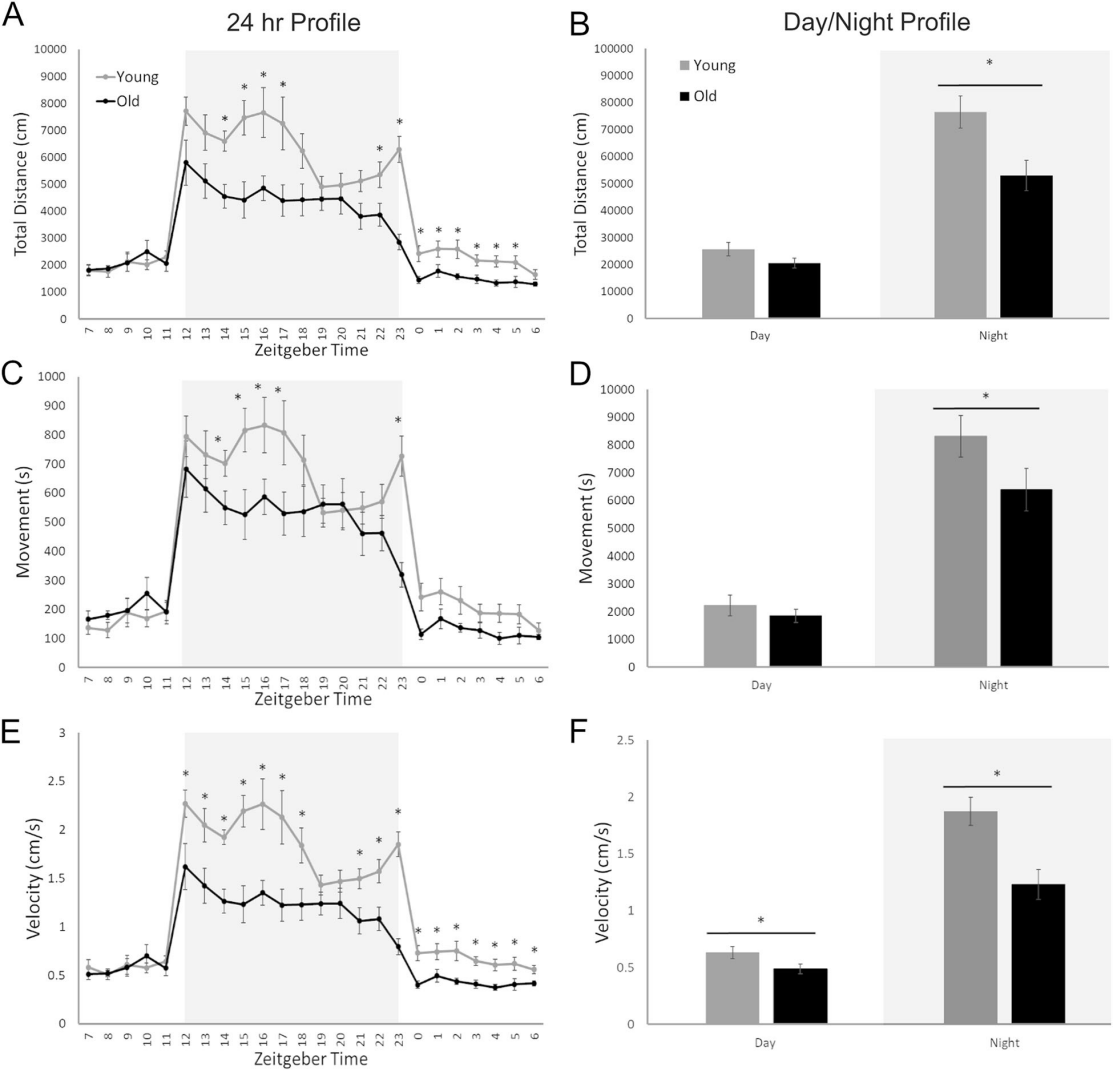
The effects of age (6 weeks vs. 18 months) on general activity levels using three variables: distance (cm), time spent in movement (s), and velocity (cm/s). Light gray blocks indicate night while white sections of the graph correspond with the day, ZT0 is light on. Distance traveled in centimeters showed significantly higher values for young mice in 24 h (**A**) and Day/Night (**B**) profiles. The 24 h profile had significantly higher distance traveled during both the night and day, however when the data were binned into 12 h night and day values, only the nighttime differences were significant. Movement time in seconds showed significantly higher values for young mice in 24 h (**C**) and Day/Night (**D**) profiles. For this variable, the timepoints with significant differences were only during the night for both 24 h and day/night profiles. Velocity in centimeters per second showed significantly higher values for young mice in 24 h (**E**) and Day/Night (**F**) profiles. Unlike the previous two variables there were significantly difference during both the night and day for 24 h and day/night profiles. *p < 0.05.

The 24-hour profile of all three general activity variables followed the same pattern: low activity during the daytime (inactive phase) and heightened activity during the nighttime (active phase). Time (F(2.867, 40.137) = 57.99, *p* < 0.001) and age (F(1,14) = 8.55, *p* = 0.011) both had main effects on total distance traveled (Fig. 1A). There was also an interaction between age and time (*F*(2.867, 40.137) = 4.887, *p* = 0.006) with different distances based on age. Young animals traveled more than older animals but only at ZT14-17 and ZT22-23. A main effect for time (F(3.606, 50.485) = 78.45, *p* < 0.001) was found for total time spent in movement (Fig. 1B) as well as an interaction between time and age (*F*(3.606, 50.485) = 4.372, *p* = 0.005) with increased time spent moving among young animals at ZT14-17 and ZT23. As both distance and time spent moving were suppressed by age, we saw the greatest effect on velocity (Fig. 1C). Time (*F*(3.270, 45.786) = 59.27, *p* < 0.001) and age (*F*(1, 14) = 13.43, *p* = 0.003) had main effects on velocity with an interaction between the two variables (*F*(3.270, 45.786) = 5.83, *p* = 0.001). These results indicate that velocity for young animals was significantly faster than older animals both at night (ZT12-18, ZT21-23) and during the day (ZT0-ZT6). Overall, significant differences between the two age groups were detected for all variables of distance, movement, and velocity.

Day/night analysis between the variables generally showed a stronger effect of age on general activity during the active phase than the inactive phase. Distance traveled (Fig. 1D) had main effects of both time (*F*(1, 14) = 117.82, *p* < 0.001) and age (*F*(1, 14) = 5.76, *p* = 0.031), and an interaction between the two variables (*F*(1, 14) = 8.55, *p* = 0.011). There was a significant difference between young and old animals only during the active phase points (*t*(14) =2.86, *p* = 0.013). Total time spent in movement (Fig. 1E) had a main effect of time (*F*(1, 14) = 187.08, *p* < 0.001) and an interaction between time and age (*F*(1, 14) = 4.85, *p* = 0.045). There was a significant difference between age groups only during the nighttime points (*t*(14) = 2.22, *p* = 0.043). Again, the greatest effects were observed with velocity, as both distance traveled and time spent moving were suppressed in older animals. Velocity (Fig. 1F) had main effects of both time (*F*(1, 14) = 137.07, *p* < 0.001) and age (*F*(1, 14) = 13.42, *p* = 0.003), and an interaction between the two variables (*F*(1, 14) = 137.07, *p* < 0.001). There was a significant difference between age groups during both the active phase points (*t*(14) = 3.55, *p* = 0.003) and inactive phase (*t*(14) = 2.14, *p* = 0.050). All variables were able to effectively demonstrate the significant differences in activity between the age groups and provide unique information about these changes.

### Daily rhythms and circadian analysis

**Fig. 2:**
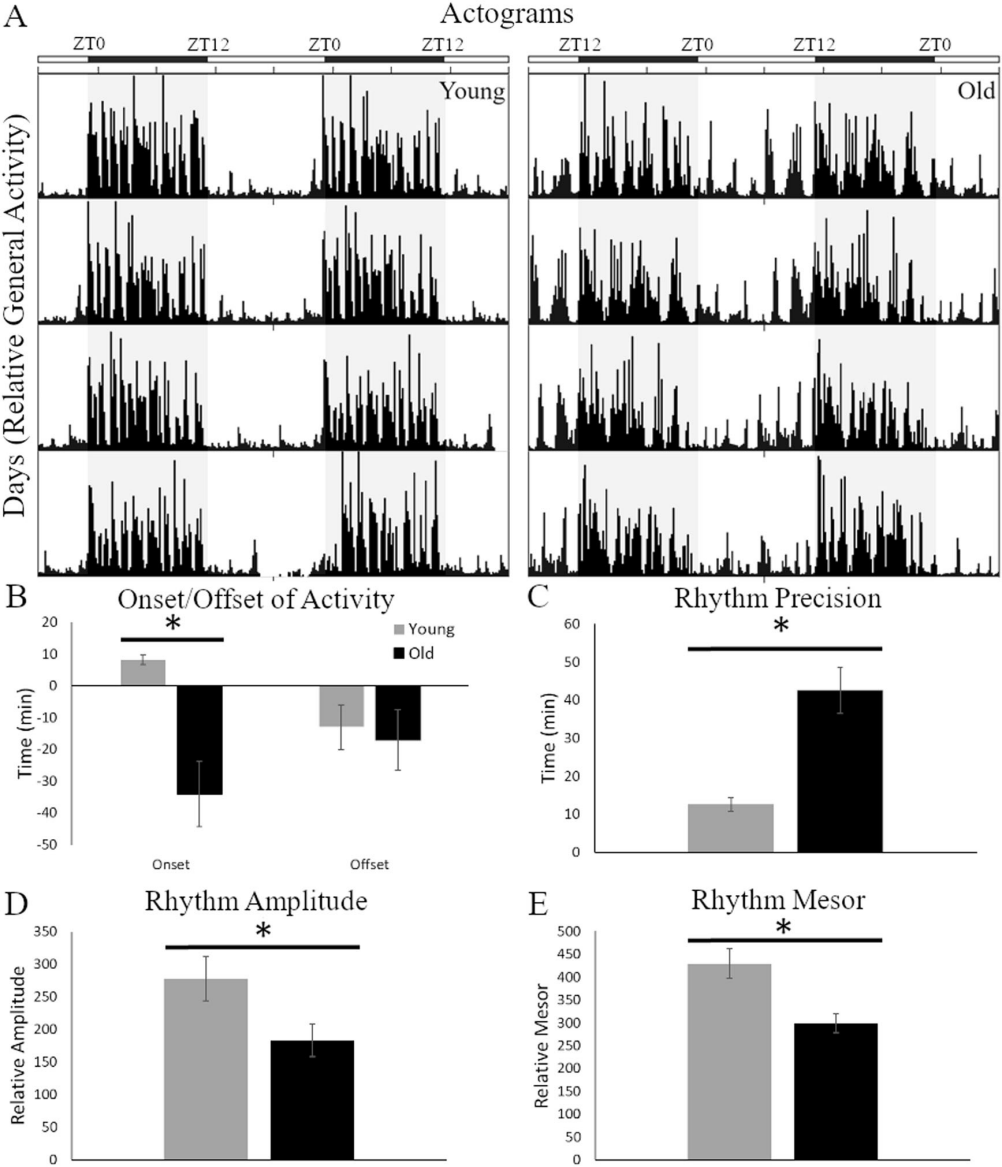
Circadian rhythms parameters examined under standard 12:12 light:dark conditions. **A** Actograms of young (left panel) and old (right panel) mice show clear differences in the circadian profiles of activity behaviors between the two age groups. Along the top of the actograms are white and black bars indicating day and night, respectively. Old mice have more diffuse activity during the day, unlike the young mice that are only active at night. **B** Onset and offsets for these mice were plotted and showed a significant difference in onset, with old animals active more than 30 min before lights off. **C** The precision of the daily rhythms was also significantly higher in old mice, indicating a less accurate internal clock. Finally, old mice showed dampened rhythms with lower amplitude (**D**) and mesor (**E**) values than young mice. *p < 0.05.

The video recording equipment and software produced data were both converted into actograms for manual circadian analysis or text files for Cosinor analysis. Representative actograms (Fig. 2A) of the two age groups demonstrate the dramatic difference in the expression of circadian parameters in 12:12 LD conditions. Onset of activity (Fig. 2B) in the older animals occurred 34.1 ± 10.5 min prior to lights off which was significantly earlier (*t*(14) = 4.02, *p* = 0.001) than younger animals that started activity after lights off, 8.1 ± 1.5 min. Offset of activity was similar between the two age groups occurring between 13 and 17 min before lights on (*t*(14) = 0.31, *p* = 0.733). Altered onset impacted the lengths of both the active (alpha, *α*) and inactive (rho, *ρ*) periods. Older animals had significantly longer α’s (*t*(14) = 2.17, *p* = 0.048) and shorter ρ’s (*t*(14) = 3.14, *p* = 0.007), making the alpha/rho ratio higher in older (1.06 ± 0.14) than younger (0.92 ± 0.06) mice and demonstrating alterations in the circadian phase and increased susceptibility to desynchrony. The precision of the onset (Fig. 2C), which represents day-to-day variability in the pacemaker period or wake-up time^32,33^, was significantly worse in older animals with a higher degree of variance between the 8 days analyzed (*t*(14) = 4.76, *p* = 0.001). Additionally, the actograms highlight the greater proportion of activity at night in older mice. When comparing the percentage of general activity during the day and night the pattern is different than the raw values above. Older mice spend significantly (*t*(14) = 1, 11, *p* = 0.032) more of their active time at night, 28.7 ± 0.02%, when compared to younger mice, 25.2 ± 0.02%.

Cosinor analysis provided information on the features of a sinusoidal wave fitted to the data and demonstrated both similarities and differences between the age groups. Variables that measure time were not significantly different between the age groups. The period of the rhythm (*t*(14) = 0.98, *p* = 0.345) in older and younger animals in 12:12 LD were both close to 24 h. Acrophase, the time of the wave peak, was also not significantly different between the two age groups (*t*(14) = 1.57, *p* = 0.139); occurred at 21.2 ± 0.6 h for older animals and 22.1 ± 0.2 h in younger animals. Variables that measure the height and strength of the sine wave were both significantly different (Fig. 2D, E), amplitude (*t*(14) = 2.23, *p* = 0.042) and mesor (*t*(14) = 3.39, *p* = 0.004), respectively. Circadian parameters in 12:12 LD examined here are clearly impacted by aging.

### Sleep behavior

**Fig. 3:**
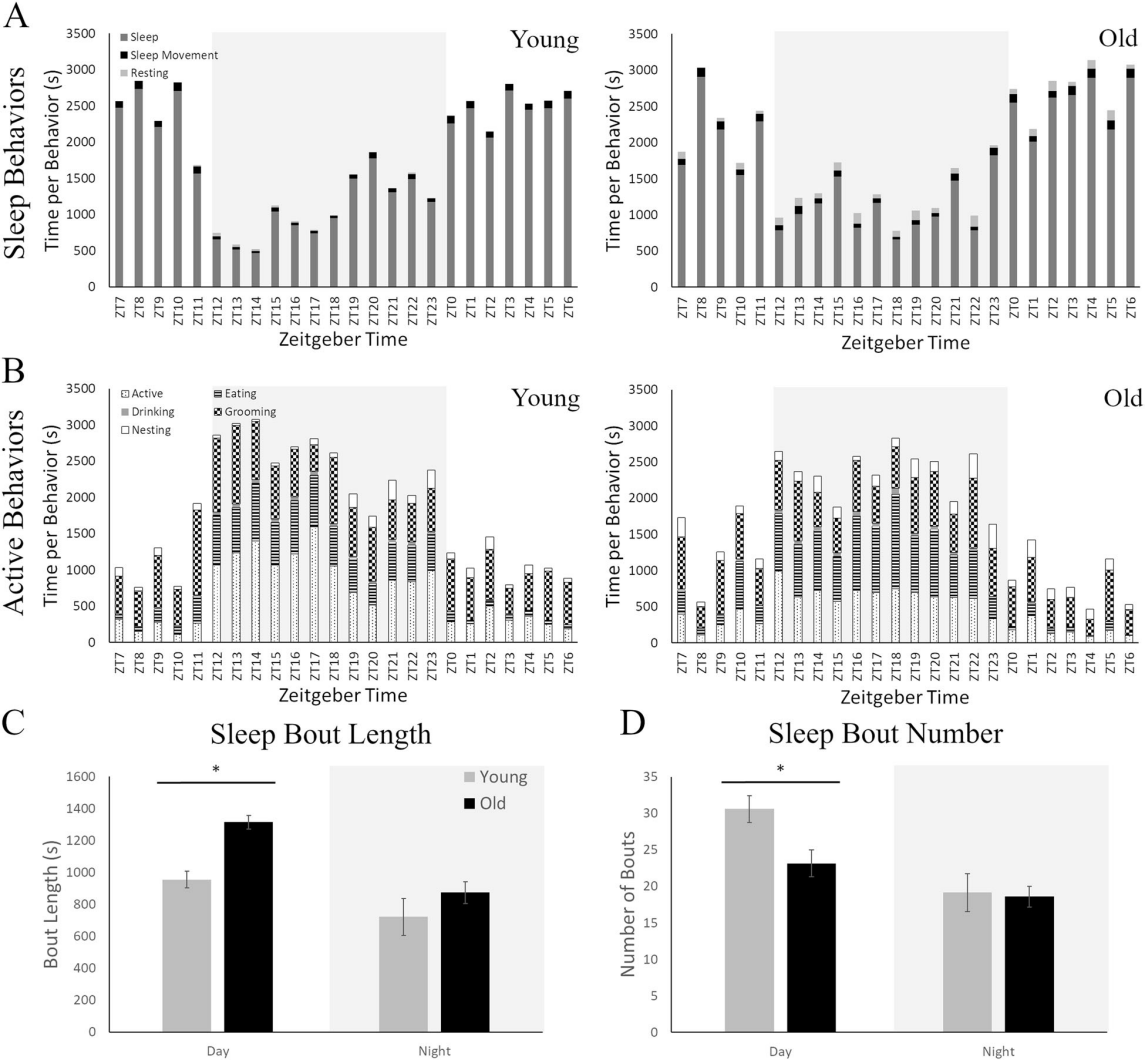
Sleep analysis of young and old mice using the manual video scoring technique. **A** Sleep-like behaviors plotted across 24 h in seconds spent for each behavior where ZT0 is lights on and ZT12 is lights off. These behaviors include sleep, sleep movement, and resting. The light gray highlighted area indicates the nighttime (active period) while white indicates the daytime (inactive period). Both young (left panel) and old (right panel) mice sleep less during the night, as mice are nocturnal animals. Young animals, however, do not display resting behaviors (light gray) while old animals have many periods of rest across the day. **B** Active behaviors plotted across 24 h in seconds spent for each behavior at the hour level. These behaviors include active (walking, climbing, rearing, and sniffing), eating, drinking, grooming, and nesting. Young animals spend more time engaging in active behaviors during the night than their older counterparts. **C** Analysis of the length of sleep bouts was significantly higher in old mice only during the daytime. **D** The number of sleep bouts showed the opposite pattern with significantly higher number in young mice, again only during the daytime. *p < 0.05.

Older and younger mice had distinct differences in the percentage of exhibited behaviors across the active and inactive periods (Fig. 3A, B). Most behaviors had a main effect of age: sleep movement, resting, grooming, nesting, and eating, or an interaction of age with time: sleep and activity (Table 1). Younger animals had higher levels of sleep, activity, and grooming, while older animals had more sleep movement, rest, nesting and eating. Drinking was the only variable to have only a time effect with drinking happening primarily in the night (active period).

**Table 1:** Statistical analysis of manually scored behavior.

	Age	Time	Age × Time	Pattern
Sleep	*F*(1, 12) = 0.013, *p* = 0.909	***F*(23, 276) = 12.310**, ***p*** < 0.001*	***F*(23, 276) = 1.691**, ***p*** = 0.027*	Younger more sleep (ZT7 & 10)
Sleep movement	***F*(1, 12) = 9.706**, ***p*** = 0.009*	***F*(23, 276) = 3.538**, ***p*** < 0.001*	*F*(23, 276) = 12.310, *p* = 0.377	Older more sleep movement
Resting	***F*(1, 12) = 44.789**, ***p*** < 0.001*	*F*(23, 276) = 0.692, *p* = 0.853	*F*(23, 276) = 0.589, *p* = 0.934	Older more rest
Active	***F*(1, 12) = 7.334**, ***p*** = 0.019*	***F*(23, 276) = 11.785**, ***p*** < 0.001*	***F*(23, 276) = 2.324**, ***p*** = 0.001*	Younger more active (ZT13, 14, and 23)
Grooming	***F*(1, 12) = 5.262**, ***p*** = 0.041*	***F*(23, 276) = 2.375**, ***p*** = 0.001*	*F*(23, 276) = 1.389, *p* = 0.114	Younger more grooming
Nesting	***F*(1, 12) = 0.6.672**, ***p*** = 0.024*	***F*(23, 276) = 1.965**, ***p*** = 0.006*	*F*(23, 276) = 0.572, *p* = 0.944	Older more nesting
Eating	***F*(1, 12) = 9.706**, ***p*** < 0.001*	***F*(23, 276) = 68.019**, ***p*** < 0.001*	*F*(23, 276) = 1.320, *p* = 0.273	Older more eating
Drinking	*F*(1, 12) = 3.867, *p* = 0.073^†^	***F*(23, 276) = 11.617**, ***p*** < 0.001*	*F*(23, 276) = 1.462, *p* = 0.083^†^	No age difference

*Bolded and statistical significance at *p* < 0.05.

^†^Trend toward significance *p* < 0.10.

The analysis of Sleep Bout number and length were also compared between the ages for both manual and automated data. For the manual scoring, there were significant differences only for the daytime period (inactive period) in both the bout length (*t*(12) = 2.499, *p* = 0.028; Fig. 3B) and bout number (*t*(12) = 2.331, *p* = 0.038; Fig. 3C). Older animals had fewer (23.14 ± 2.60) but longer (1314.07 ± 115.64 s) bouts than young animals (number:30.57 ± 1.83; length: 954.77 ± 52.67 s). Automated scoring had the same trend but only had a significant difference in length (*t*(12) = 2.531, *p* = 0.026) and a trend for bout number (*t*(12) = 1.897, *p* = 0.082) during the day. Interestingly in the automated data, older mice spent more time sleeping outside of the times defined as bouts, which was classified as “Resting” in manual scoring, compared to young animals (*t*(12) = 3.100, *p* = 0.009). Sleep behavior is different between the two ages, the novel system replicates previous findings of sleep quantity and bout number but also provides further data on additional behaviors, such as grooming, nesting and eating.

### Masking of activity to night-time light

**Fig. 4:**
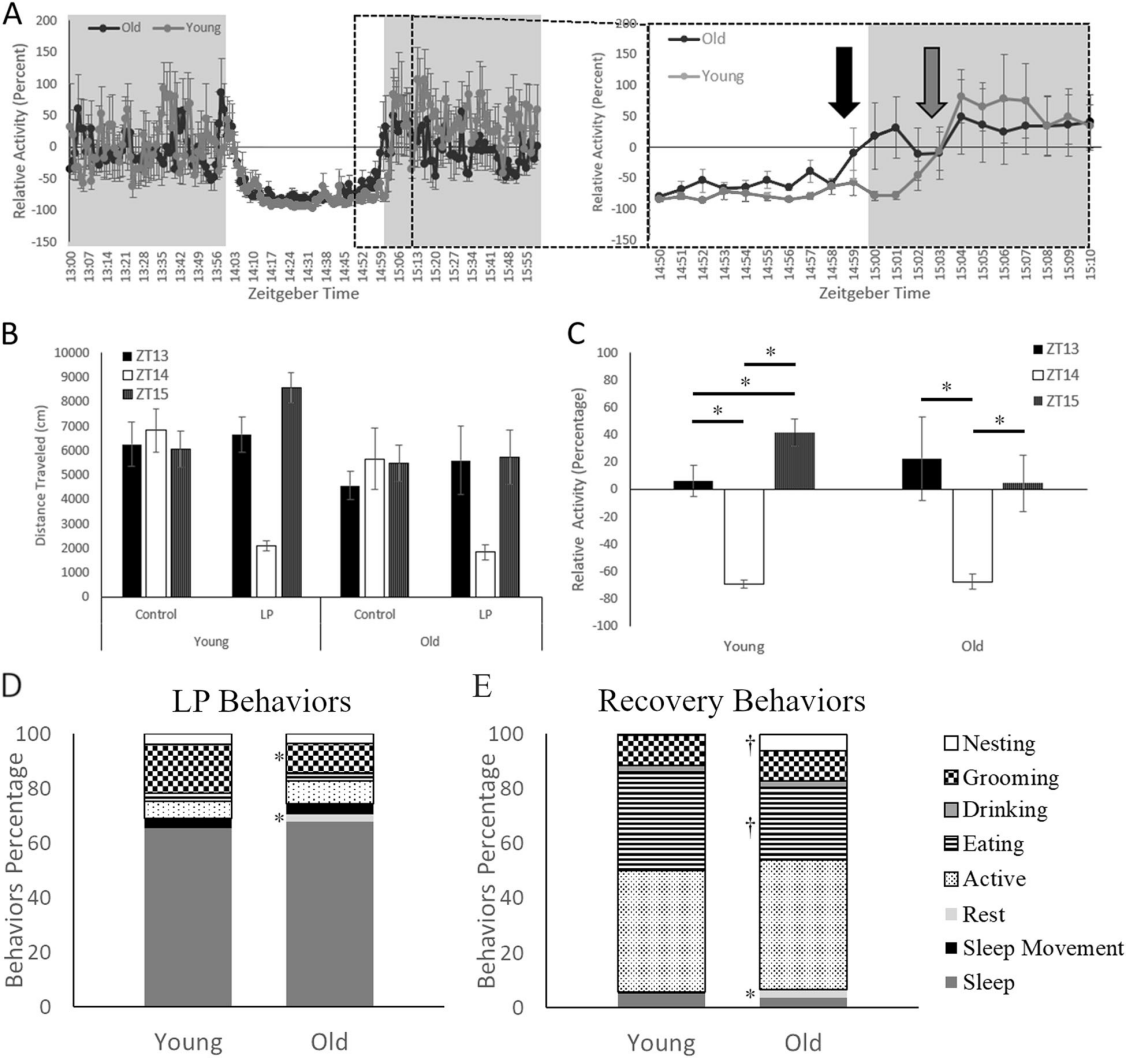
The masking effects of nighttime light exposure given at ZT14 between the age groups. **A** A plot of before, during, and after the masking pulse spanning three hours of activity in one-minute bins (Left Panel). The gray area indicates the hours before and after the light pulse (LP), while the white indicates light exposure. The inlet highlights the transition period 10 min before and after the return to darkness as animals return to baseline activity levels (Right Panel). Young animals in light gray recover from the LP after being returned to darkness (Gray Arrow) while the older animals in black do not sustain activity the entirety of the LP (Black Arrow). **B** Activity for animals plotted at the hour level on a control day, where no LP is given, did not differ across the 3 h between either young or old animals. However, both ages had dramatic differences in activity levels on the LP day. **C** Statistical comparisons of activity levels, corrected to control day levels as a relative percentage, indicate significant suppression of activity during LP and heightened recovery levels in young mice at ZT15. **D** and **E** Video analysis during LP (**D**) and Recovery (**E**) demonstrated alterations in time spent engaging in different behaviors between the age groups. Resting was significantly greater for older animals during both times, while grooming was reduced only during the LP. Old animals also had a trend in significance for higher nesting and lower eating levels during recovery. *<0.05, ^†^*p* < 0.1.

Masking differences between the age groups is hinted at in the general activity and circadian data, as older animals have higher daytime activity and activity onsets that begin during the light phase. When the masking pulse data was plotted at the minute level over the three hours (1 h before the LP, during the LP and 1 h Recovery), mice of all ages behaved similarly at the baseline hour with averages around 0% relative activity (Fig. 4A, Left). During the hour of LP, animals in both had suppressed activity within the first 5 min and suppression was identical between the old and young groups until the final 10 min of the pulse and the beginning 10 min of recovery (Fig. 4A, Right Inset). During this time older animals appear to no longer suppress activity from the LP 1 min before lights out with activity levels returning to 0%. Young animals, on the other hand, took 4 min in complete darkness to resume baseline levels of activity. Interestingly, young animals appeared to rebound differently to the LP in the recovery hour with heightened activity levels.

Gross total distance traveled for the LP day and the previous day (control) was plotted by the hour to statistically examine the phenomena (Fig. 4B). Control times did not have significant main effects of age (*F*(2, 28) = 13.42, *p* = 0.564) or time (*F*(2, 28) = 0.638, *p* = 0.536) and no interactions (*F*(1, 14) = 1.621, *p* = 0.224). The lack of significance indicates that activity is the same for all the ages and times on the control day. Relative activity levels were calculated by dividing each hour by the same hour on the control day and multiplying by 100 to create a percentage (Fig. 4C). One-way ANOVAs showed significant difference in activity at the three time points for both young (*F*(2, 14) = 63.94, *p* < 0.001) and old (*F*(2, 14) = 7.073, *p* = 0.008) animals. Young animals had a different pattern, with higher activity on the hour of recovery than the baseline hour prior to the LP (*t*(7) = 3.464, *p* = 0.01).

Manual scoring of behavior during and after the LP for eight animals (four mice/group) showed no significance during the light pulse time for the combined active (*t*(6) = 1.829, *p* = 0.117) and inactive (*t*(6) = 1.828, *p* = 0.117) variables. Only rest (*t*(6) = 2.584, *p* = 0.042) and grooming (*t*(6) = 3.271, *p* = 0.017) were significantly different between the age groups at the individual variable level. Older animals rested more (+2.75%) and groomed for less time (−7.32%) than younger animals. During the recovery hour, there was a trend toward significance for both the combined active (*t*(6) = 2.292, *p* = 0.062) and inactive (*t*(6) = 2.076, *p* = 0.083) variables, with younger animals active for a greater percentage of the time (+1.43%). Again, rest was the only variable that was significantly higher for older animals (*t*(6) = 2.847, *p* = 0.029). Together, these data suggest masking is directly impacted by aging more clearly during the recovery from the exposure to light.

### Long-term alteration in the non-image forming visual pathway

#### Histology: retinal and brain

**Fig. 5:**
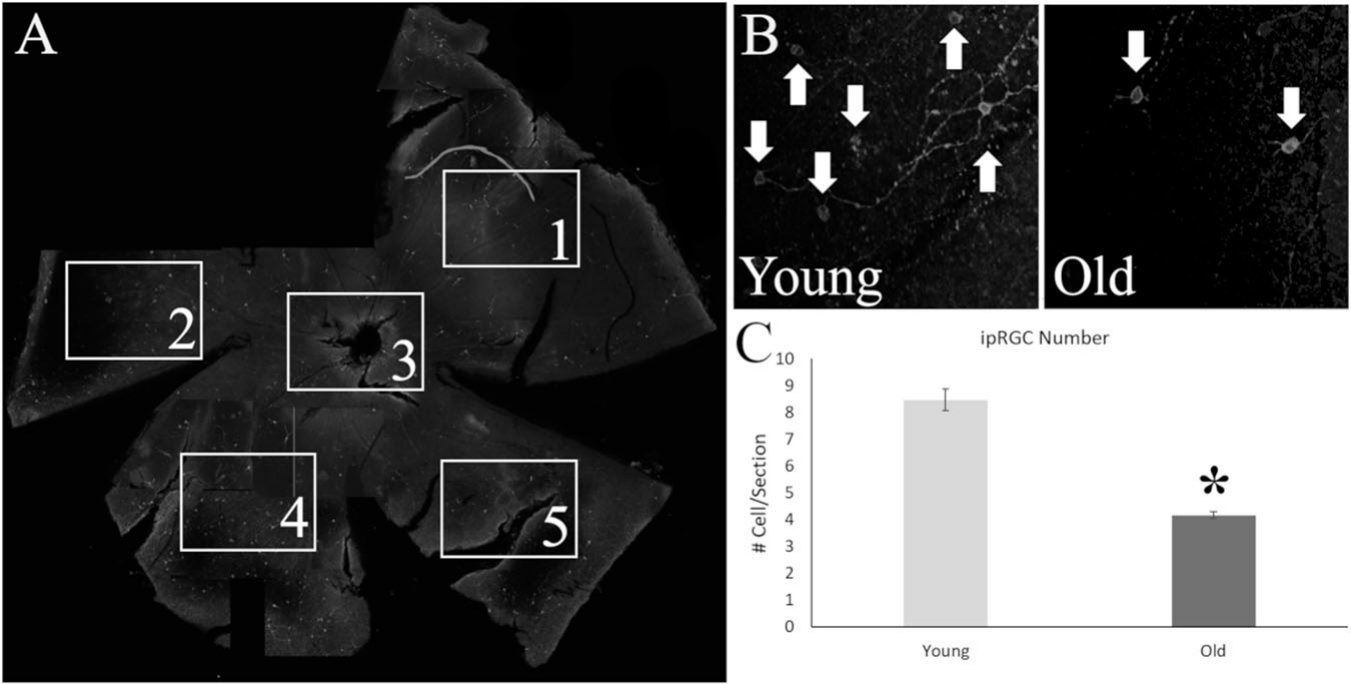
Histological analysis of intrinsically photosensitive retinal ganglion cells (ipRGC) from the retinas of old and young mice stained for melanopsin. **A** Whole mount retinas of the mice were divided into four petals, one image for counting was taken for each petal and from a central location. **B** The number of total melanopsin positive cells (White Arrows) were lower in old mice (Right Panel) than in their younger counterparts (Left Panel). **C** Statistical analysis of the number of cells per section demonstrated a significant difference between the age groups, with a 50% reduction in number of cells in older mice. *p < 0.05.

**Fig. 6:**
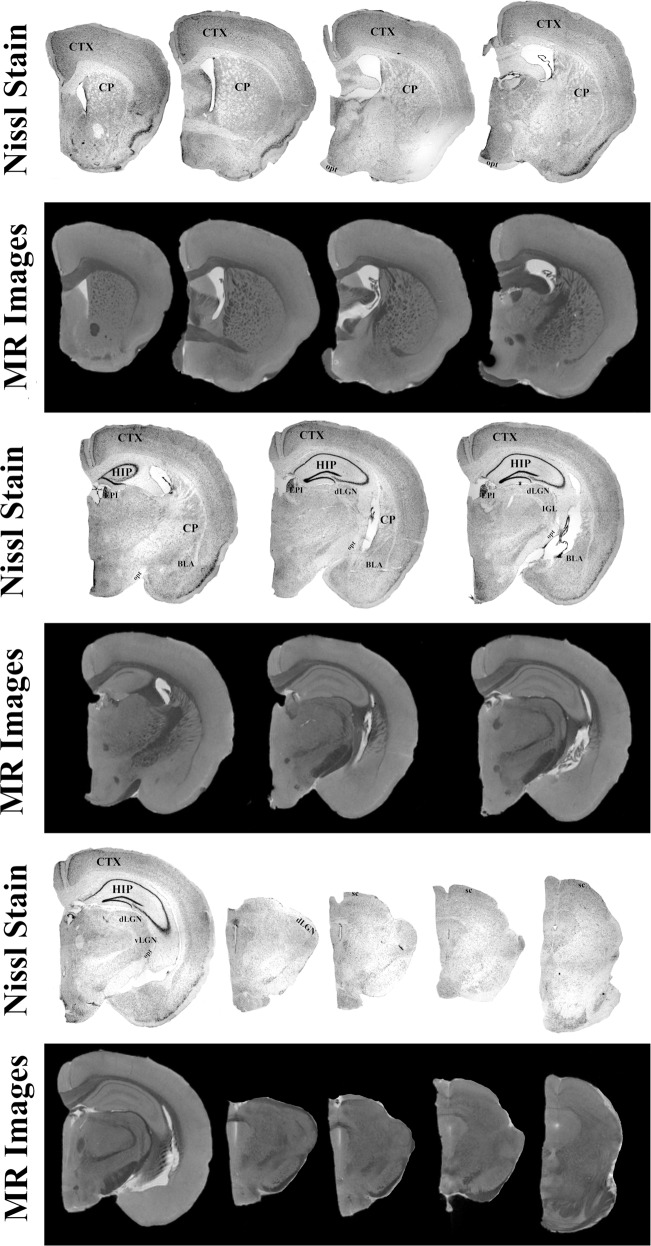
High-resolution T1 weighted magnetic resonance images compared to Nissl stained brain sections from one representative mouse. Structures observed within the stained section are clearly identified within the scans. These structures include the cortex (CTX), caudate nucleus (CP), hippocampus (HIP), habenula (EPI), basolateral amygdala complex (BLA), optic tract (opt), superior colliculus (SC), and lateral geniculate nucleus (LGN). The greater resolution (32 µm) utilized in this manuscript allowed for the identification of subregion of the LGN, ventral (vLGN) and dorsal (dLGN) components.

Retinas of both young and old animals were stained for melanopsin and counted using confocal microscopy (Fig. 5A). On average, the total number of ipRGCs in the 18 month old animals was 51% less than 6 week old animals (Fig. 5B) and was significantly different from one another (*t*(6) = −10.182, *p* < 0.001; Fig. 5C). Figure 6 shows the comparison of Nissl staining to MRI for one representative animal. All regions of interest are clearly visible, validating the use of MRI for the quantification of the volumes of these regions. Higher resolution MRI, 33 µm vs. 50 µm, allows for further subdivision than our prior publication^34^ of the geniculate complex into dorsal and ventral components. Additionally, as described below, we analyzed regions involved in various learning and memory also visible in our MR images, including the caudate nucleus, cerebellum, and basolateral amygdala.

### Ex vivo MRI volume analysis

**Fig. 7:**
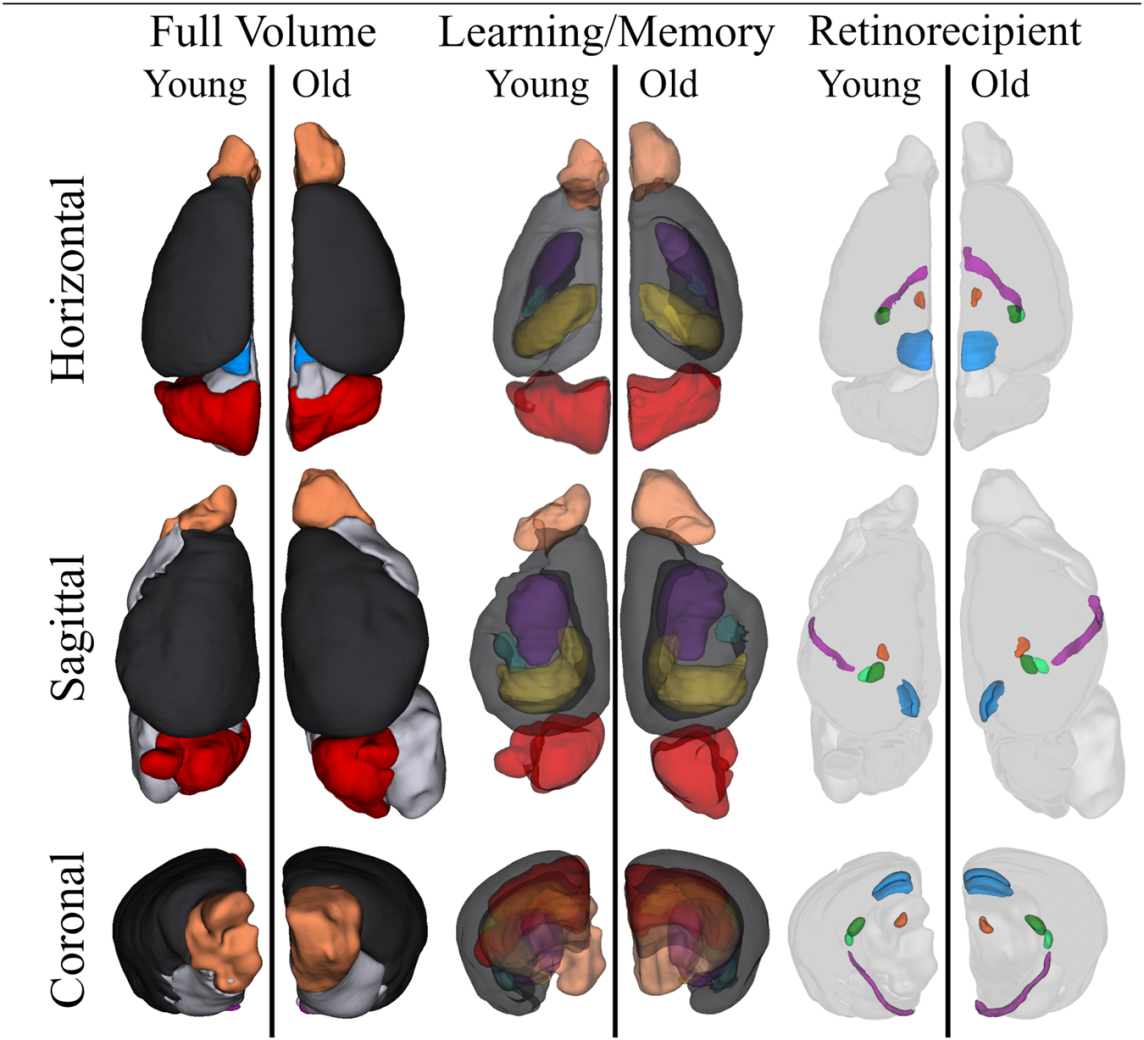
Three-dimensional volume rendering of learning/memory and retinorecipient non-image forming visual regions in young and old mice. Volumes for the cortex (black), olfactory bulb (light orange), hippocampus (yellow), amygdala (teal), caudate nucleus (dark purple), cerebellum (red), superior colliculus (blue), geniculate complex (dorsal, dark green; ventral, light green), habenula (dark orange) and the optic tract (light purple) are shown in three orientations. The brains of the two age groups are represented in identical proportions to demonstrate the relative size differences between visual structures. Most notably, a greatly enlarged optic tract is observed within older mice.

Total brain volumes between young and old animals were not statistically different between the two age groups (Table 1). Young animals had an average brain volume of 218.73 ± 9.9 µm, while older animals had slightly larger brains, 240.18 ± 7.14 µm. Comparing the 3D volume maps, there are regions that appear to have visible differences between the ages in both learning/memory and circadian visual circuits (Fig. 7). For the learning/memory regions, the hippocampus and cerebellum are clearly smaller in the older animals; the olfactory bulb, on the other hand, appears smaller in the younger animal, however, this is an artifact of damage sustained to the brain of the representative mouse during extraction. While the literature often only focuses on the hippocampus when interrogating changes in learning and memory, here we are including other critical structures that are often overlooked. When comparing the relative differences between the age groups, the cortex (−12%), hippocampus (−26%), and cerebellum (−20%) all had significantly decreased volume sizes in older mice. The amygdala also had a decrease in old animals (−25%) but the difference was only trending toward significance (Table 2). For the circadian visual system, the most striking difference in the 3D volume (Fig. 7) was the larger optic tract observed in older mice. When comparing the relative differences between the age groups, the optic tract (+47%), superior colliculus (−19%), habenula (−30%), and dorsolateral geniculate nucleus (−25%) were all significantly different between the groups. The superior colliculus (SC), habenula (EPI), and dorsal lateral geniculate nucleus (dLGN) were smaller in the older animals, while the optic tract was larger. These data demonstrate that age impacts the total volume of areas critical for learning and memory as well as sleep and circadian rhythms, and are the first to describe this long-term atrophy in the non-image forming visual system.

**Table 2:** Quantification of regions of interest MR images.

	Age	Volume (mm^3^)	Statistics	Significance	Percentage (%)	Statistics	Significance
Whole	Young	218.73 ± 9.9	*t*(8) = 1.759	*p* = 0.117			
	Old	240.18 ± 7.14					
*Learning/Memory regions*							
CTX	Young	71.03 ± 1.96	*t*(8) = 0.759	*p* = 0.470	**32.51** ± **0.48**	***t*(8) = 3.277**	***p*** = 0.011*
	Old	68.63 ± 2.48			**28.66** ± **1.06**		
OB	Young	9.43 ± 0.56	*t*(7) = 1.378	*p* = 0.211	4.11 ± 0.21	*t*(7) = 0.006	*p* = 0.995
	Old	10.29 ± 0.44			4.32 ± 0.26		
HIP	Young	**12.37** ± **0.46**	***t*(8) = 3.800**	***p*** = 0.005*	**5.67** ± **0.26**	***t*(8) = 4.226**	***p*** = 0.003*
	Old	**10.09** ± **0.38**			**4.23** ± **0.22**		
CP	Young	12.09 ± 0.53	*t*(8) = 0.513	*p* = 0.622	5.53 ± 0.20	*t*(8) = 1.137	*p* = 0.288
	Old	12.45 ± 0.45			5.20 ± 0.21		
CB	Young	**31.44** ± **1.02**	***t*(7) = 3.556**	***p*** = 0.009*	**14.06** ± **0.56**	***t*(7) = 3.787**	***p*** = 0.007*
	Old	**27.07** ± **0.63**			**11.32** ± **0.41**		
BLA	Young	2.33 ± 0.32	*t*(8) = 1.252	*p* = 0.246	0.32 ± 0.04	*t*(8) = 2.060	*p* = 0.073^†^
	Old	1.92 ± 0.11			0.24 ± 0.01		
*Circadian visual regions*							
opt	Young	**2.07** ± **0.04**	***t*(4) = 5.814**	***p*** = 0.004*	**0.27** ± **0.01**	***t*(4) = 3.484**	***p*** = 0.025*
	Old	**3.32** ± **0.16**			**0.40** ± **0.03**		
dLGN	Young	**2.44** ± **0.12**	***t*(8) = 2.525**	***p*** = 0.036*	**0.33** ± **0.03**	***t*(8) = 2.975**	***p*** = 0.025*
	Old	**2.03** ± **0.11**			**0.25** ± **0.01**		
vLGN	Young	0.79 ± 0.05	*t*(8) = 0.161	*p* = 0.876	0.11 ± 0.01	*t*(8) = 0.878	*p* = 0.406
	Old	0.8 ± 0.05			0.10 ± 0.004		
EPI	Young	1.05 ± 0.08	*t*(8) = 1.928	*p* = 0.090^†^	**0.14** ± **0.02**	***t*(8) = 2.557**	***p*** = 0.034*
	Old	0.83 ± 0.08			**0.10** ± **0.01**		
SC	Young	4.63 ± 0.11	*t*(8) = 1.644	*p* = 0.139	**0.63** ± **0.02**	***t*(8) = 3.072**	***p*** = 0.015*
	Old	4.12 ± 0.28			**0.51** ± **0.03**		

*CTX* cortex, *OB* olfactory bulb, *HIP* hippocampus, *CP* caudate nucleus, *CB* cerebellum, *BLA* basolateral amygdala, *opt* optic tract, *dLGN* dorsal lateral geniculate nucleus, *vLGN* ventral lateral geniculate nucleus, *EPI* habenula, *SC* superior colliculus.

*Bolded and statistical significance at *p* < 0.05.

^†^Trend toward significance *p* < 0.10.

## Discussion

This study demonstrated that aging greatly impacts (1) behavioral expression of general activity, circadian rhythms, sleep, and masking to night-time light and (2) the morphological structures of the retina and brain important for the regulation of sleep and circadian rhythms. Using a novel video tracking system, we accurately monitored detailed information about the activity patterns, including distance traveled, time spent moving, and velocity, as well as the identification of complex behaviors, such as sleep, grooming etc., at levels comparable to a that of a trained human scorer. These complex behaviors have not been explored in the same capacity in previous publications, as it would require more even more time to process the data for a human scorer. The automated system significantly reduces the total man hour needed to acquire the data, with one scorer taking 6 h at 4× speed to quantify the behavior of one animal for 1 day. The automated system scores the data in real time as the videos are recorded. Our findings further highlight the impact of aging on the non-image forming visual systems; with reductions in number of retinal ipRGCs and decreased brain volumes in older mice. These findings suggest that disruptions of sleep and circadian rhythms are partially attributed to impaired input pathways, which lead to less entrainment information activating regions of the brain important for promoting sleep and circadian behaviors. The decreased sensitivity of these regions to light information from the retina caused by age may predispose older individuals to developing treatment-related symptoms, suggesting the need to understand the effects of treatment on these pathways.

Sleep disturbances are more common in patients with chronic diseases such as cancer^35^, and cancer treatments, like radiation, can contribute to the development of symptoms^36^. Older individuals are more sensitive to developing these sleep issues after treatment^37^ and demonstrate weaker rhythms compared to young controls in the healthy population^6^. Behavioral alterations in sleep and circadian rhythms in our mouse model are similar to those described in aged humans and other rodent models. Here we validate the use of an automated video system, video recording cages and software, for the analysis of sleep and circadian rhythms and explore the masking responses of older animals for the first time. To our knowledge, only one study has implemented the use of the software to plot activity across 24 h for mice but did not investigate detailed circadian parameters^38^. The study collected telemetry data and suggested that the surgical implants required were more invasive than video, but did not compare the results to sleep calculated by the Mouse Behavioral Module. Video analysis also encourages more natural behavior than the use of traditional running wheels, which can alter rhythms as they are addictive^39,40^. Our study easily detected the decrease in general activity and dampened amplitude of daily rhythms in older mice that is observed with traditional activity detection^41^. From the actograms we generated, precision of activity onset was calculated and again matched the results of the previous literature^15,42^. Circadian rhythms were therefore easily analyzed with the automated data collected of the video recording cages and software and duplicated the results of traditional methods. In our hands, sleep detection with the automated system was equivalent to manual scoring by an experienced scorer. The analysis was able to easily detect differences in both sleep quantity between older and younger mice, and the alteration in bout length and number caused by age.

We also explored the impact of age on the masking responses to light using the video system in our mouse model. Masking is the immediate response to the presentation of a stimulus, such as light, that can interfere with the examination of intrinsic circadian rhythms^43^. However, these behaviors play a crucial evolutionary role in promoting behavior during the appropriate time-of-day; for nocturnal mice, light will induce sleep, while for diurnal humans, it promotes activity^44^. Patients that experience sleep disturbances after treatment often report heightened daytime sleep or hypersomnolence, which should not occur if masking is promoting activity during the light phase. The suppression of activity in our animals was similar between age groups for the hour of light presentation during the night. Other studies that have examined the pupillary light reflex, which uses the same visual pathway as masking, reported similar findings (no or little significant differences between ages^45,46^ in humans and mice^30^. Although in our data, older animals did recover from the LP before they were returned to darkness and there was a large significant difference in the degree of the recovery response after LP between the ages. There is only one other paper that explores masking effects of light in older mice reports similar findings, as they show only two significant age effect for proportion of time asleep following light pulse and speed of waking in B6N mice^47^. That study however exposes mice to a LP at ZT16 and only compares middle aged (5 month) to older (20 month) mice. During the normal LD cycle, we also observed higher daytime activity and activity onsets that begin during the light phase, all suggesting differences in the degree of masking in the older population. Further investigation into the effects of age on masking are merited including the timing of LP and intensity or duration of the light pulse. These findings also pose the interesting question of why masking behavior to light appears to be better maintained in older mice than circadian rhythms. Overall, behavioral activity, sleep and masking differences between the age groups are clearly detected with the system. All three behaviors are dependent on the non-image forming visual pathway prompting our investigation of this system in the retina and brain.

The impact of oncologic therapy can manifest as changes in brain structure volumes; however, studies have only focused on learning and memory regions. Specifically, hippocampal volume loss due to cranial irradiation is a commonly used predictor of cognitive disfunction^48^. The negative effects of cranial irradiation have encouraged the development of treatment plans that spare damage to the hippocampus to avoid negative cognitive outcomes^49^. Our animals showed decreases in size within the hippocampus, cortex, and cerebellum in the old group, which is similar to previously published findings in aged humans^45–52^. We examined the importance of other regions that regulate sleep and circadian rhythms in this same manner. The non-image forming visual system is key in regulating circadian rhythms, sleep and masking behaviors; functioning to transmit important light information^27–29^ and driving behavior directly^53–57^. Others have shown that the SCN and IGL are less activated by light in older animals^58,59^, here we demonstrate that these decreases in activation from the deterioration of retinal ipRGC have long term consequences in brain volumes. In our animals, we found significant differences in sizes of the opt, SC, dLGN, and EPI between our 7 week and 18-month-old mice. Previous literature has demonstrated that damage to retinal projections leads to decreases in volume of these regions in the healthy organisms^60–62^. Our study demonstrated marked decreases in the number of ipRGCs observed within the retina of older mice, approximately halving the number of cells and therefore reducing the possible signal generated within the eyes. Since light information that regulates circadian rhythms, pupillary light reflex and masking is only transmitted directly by ipRGCs or through the ipRGCs as a conduit from the rod/cones^27–29^, these findings suggest that brain regions receiving light information atrophy as older animals lose ipRGCs critical for signaling within the non-image forming visual pathway. The opt was the only region that showed a dramatic increase in size with age, however, prior studies had demonstrated that older animals develop gliosis within the optic tract^63,64^. Therefore, the tract could be enlarged due to inflammatory responses or other changes within the structure as myelin degenerates across age and does not indicate increased projections. Clearly, aging impacts the size of these regions and the expression of circadian/sleep patterns generally; it may therefore benefit the patient populations with progressed age to protect these regions during medical therapy.

Overall, our work established a novel video system capable of visualizing behavioral changes of sleep and circadian rhythms beyond those traditionally described in the literature, and for the first time demonstrated the long-term atrophy of brain regions during the normal aging process in a mouse model. This provides an important insight into the alterations that predispose older individuals to developing circadian and sleep dysfunction with additional environmental stressors. This work showed that the aged circadian visual system, which delivers important input to the SCN and downstream non-image forming visual regions, is affected at the level of the retina and more importantly that these alterations within the retina causes by age lead to atrophy within the brain. Our previous publications have highlighted the importance of some of these downstream regions in driving the normal activity and sleep patterns of animals^34,65–67^. Behaviorally, daily activity is greatly suppressed in older mice, characterized by slower movements and shorter distances. Additionally, during masking light exposure, older mice do not sustain suppressed activity for the complete LP duration and have lower rebound in activity upon return to darkness. Circadian parameters in older mice also demonstrated a dampening and lowered precision in daily rhythms. Finally, younger animals slept less during daytime (ZT7 and 10) and have shorter duration but increased number of sleep bouts. Overall, we replicated the previous behavioral findings with a modern technique at a more granular level, which included the description of other complex behaviors. Our results demonstrate the effective use of a novel video monitoring system for quantifying circadian rhythms and sleep in mice and validates the ability of structural MRI to monitor intraspecies changes in brain volume caused by age. Aging clearly impacts circadian rhythms and sleep behavior, and these findings are correlated to the atrophy found within the non-image forming visual system. Ultimately, monitoring these brain changes will be critical for understanding sleep disturbances in patients and will provide potential mechanisms for intervention. Cranial irradiation has already been directly linked to the atrophy of brain regions important for learning and memory and decline in cognitive function for brain tumor patients^48,49^. Our future work will investigate the sleep disruption after cancer treatments, such as cranial irradiation, and will integrate volume monitoring of the non-image forming visual system^68^, as atrophy of these regions are clearly linked to behavioral changes in aged mice.

## Methods

### Animals and housing

Sixteen adult, male C57BL/6 mice were obtained from Charles River Laboratories (CRL, Dublin, VA). Only males were used in this study, however, future work should include both sexes. Animals were singly housed in PhenoTyper® 3000 cages (Noldus Information Technologies; Wageningen, Netherlands) and monitored continuously using the Ethovision XT 14 Software (Noldus Information Technologies). Food and water were provided ad libitum and lights were on a 12:12 Light:Dark (LD) schedule with intensities ranging from 0 to 60 lum/ft^2^. Experimental procedures were approved by the Institutional Animal Care and Use Committee at the National Cancer Institute and followed the National Institute of Health Guide for the Care and Use of Laboratory Animals (NIH Publications No. 80-23) revised 1996.

### Experimental design

Two age groups were compared within the experiments, a young group (7 week, *n* = 8) and an old group (18 months, *n* = 8). Young animals were acclimated for 7 days upon arrival from CRL, while old mice were aged in house to ~18 months of age. We compared several important behaviors impacted by age using the novel PhenoTyper® video system: general activity daily rhythms, sleep-like behavior, and masking responses to nighttime light (Supplemental Fig. 1). To examine sleep-like behavior, we used the Automatic Mouse Behavior Recognition Module (Noldus Information Technologies), which is a computer automated program designed to score behaviors. To validate the accuracy of the software, one blinded scorer (DSM) manually identified sleep-like behavior, grooming, eating, and drinking and compared it to the results obtained from the software on one representative day (day 4). General activity was examined across days 3–10. Masking to a 1-h pulse of light was measured on the night of the 11th day at ZT14. The effects of age on the input system that regulates the circadian and masking response were examined by measuring the number of ipRGCs within the retina of all mice, and determining the relative size of regions of the brain receiving ipRGC projections using anatomical MRI and histology.

### Behavioral analysis

To examine general activity rhythms, animals were recorded in the PhenoTyper® 3000 cages for 10 days, the last 8 days were used to compare daily rhythms in general activity between the ages. Total distance traveled, movement time, and velocity were examined across 24 h in 1-h intervals and day/night levels to compare the profiles of activity between groups. Using the raw 6-min level data of distance traveled, Cosinor analysis (Roberto Refinetti’s Circadian Cosinor Software, http://www.circadian.org/main.html) determined the period, amplitude, mesor, and acrophase of the daily rhythms for all animals. To further probe circadian variables for activity, actograms of total distance traveled in 10 min bins were generated for all animals and scored for activity onset and offset by two blinded scorers (DSM and DY). Phase angle of entrainment was calculated from the difference between the activity onset and lights out. Precision was defined as the variability in the phase angle of entrainment across the 8 days of activity.

Video analysis of behaviors was scored for one day (day 4) across 24 h by a blind observer (DSM) and verified by second observer (DY). To ensure clear video monitoring of behavior, mice were only given limited nesting material and no bedding or shelter during recordings. The observer scored sleep-like behavior, sleep movement, rest, activity, grooming, nesting, eating and drinking (Supplemental Video 1). Sleep-like behavior was defined as complete immobility in one position for greater than 1 min, however if animals would adjust their bodies slightly but then continue to maintain immobility in the new position this was coded as sleep movement. Rest was defined as immobility with small movements, often of the head, and occurred outside of the nesting material. During sleep-like behavior, number of sleep bouts and total bout duration were calculated, a bout was defined as the sum of all 1-min bins that were greater than 50 s and occurred sequentially for a least 3 min. Activity was comprised of walking, climbing, rearing, and sniffing and was characterized by large movements in location within the cage. Total quantities of sleep behavior were compared across 24 h, and during the active and inactive periods to the values compared to the numbers generated by the Automatic Mouse Behavior Recognition Module (Noldus Information Technologies).

Masking behavior was tested on the 11th night of the experiment. A light pulse (LP) was given at the timepoint zeitgeber time (ZT) 14, 2 h after lights off, the optimal time established in mice to observe clear light-induced masking behavior in general activity^67^. One-hour pulses of 60 lum/ft^2^ light originated from overhead room lighting at the same intensity as experienced during the regular light/dark cycles, intensity was continually measured using the HOBO light-level logger pendant (MX2202, Onset Computer Corporation, MA) placed at cage level. Data were plotted in 1-min bins to quantify the rate of initial response to light presentation and rate of recovery when returned to darkness. Total activity in 1-h bins were measured during the hour of the LP and the hour after mice were returned to darkness. These values were corrected to the levels of activity observed during the same hours on a control day and are presented as relative percentage of activity. Video analysis of behaviors were scored during the LP hour and the hour after, and the percentage of different behaviors were plotted as bar graphs.

### Ex vivo anatomical MRI

At the completion of the behavioral experiments, animals acclimated for 3 days and then were sacrificed via cardiac perfusion using a 4% paraformaldehyde solution. Brains were post-fixed for 4 h and then transferred into 0.1% gadolinium-doped PBS solution (Magnevist, Bayer, NJ) for a minimum of 48 h prior to scanning. Before scanning, brains were halved and placed in a 10 mm tube filled with Flourinert (3 M Company, MN). The tubes were secured into 14.1 T/4 cm Bruker microimaging scanner with a MicWB40 probe (Bruker Corporation, MA) and imaged using a FLASH sequence with the following parameters: TE = 5 ms, TR = 50 ms, FOV = 1.6 × 0.8 × 0.8 cm, resolution = 32 × 32 × 32 µm, Averages = 10 and total scan time = 8 h 49 m. Images were converted into DICOM files and analyzed for volume differences using FIJI Measure tool^69^ as previously described^34^. In brief, three components of the retinohypothalamic tract were measured in both young and old mice: optic tract (opt), superior colliculus (SC), and lateral geniculate nucleus (LGN) subsections into ventral (vLGN), dorsal (dLGN). Regions of the brain associated with learning and memory were also quantified and included the hippocampus (HIP), caudate nucleus (CP), olfactory bulb (OB), cortex (CTX), cerebellum (CB), and basolateral amygdala complex (BLA). Total brain volume for each animal was used to correct volumes of all regions which eliminates the effects of possible overall size differences between young and old animals. Values produced are therefore a relative percentage. Volume renderings for figures were generated using the 3D Slicer 4.8 program (https://www.slicer.org/)^70^.

### Retinal and brain histology

Retinas were extracted from mouse eyes after post fixation for 1 h and stained for melanopsin, as previously reported^66^. In brief, whole eyes were removed from perfused mice and were place in a 5% PFA solution for 1 h. Using a Leica dissecting microscope, retinas were microdissected from the sclera and stored in PBS at 4 °C until staining. Whole retinas were rinsed with 1% hydrogen peroxide solution and then washed three times in 0.3% Triton-X in 0.01 M PBS. Retina were blocked for 1 h at room temperature in normal donkey serum (Jackson ImmunoResearch Laboratories, West Grove, PA) and then incubated for 72 h at 4 °C in rabbit anti-OPN4 primary antibody (1:2000, Invitrogen, Carlsbad, CA). Sections were washed three times with 0.3% Triton-X in 0.01 M PBS and then incubated for 1 h with a fluorescently tagged secondary antibody, Cy3-conjugated donkey anti-rabbit (1:200, Jackson ImmunoResearch Laboratories, West Grove, PA). Whole mount retinas were placed on glass slides and coverslipped with ProLong diamond antifade mountant (Life Technologies Corporation, Eugene, OR). After 24 h, slides were imaged with a Zeiss confocal microscope (LSM 700) at five locations across each retina at 20× magnification. Using the Image J cell count plug-in, each image was counted for number of total OPN4 + cells.

Brains were flash frozen in Optimal Cutting Temperature media and sectioned on a cryostat (Leica) at −19 °C with 25 µm thickness. Every third section was mounted on gelatinized slides and dried overnight before staining with thionin as described in Gall et al.^66^ to delineated regions of interest, including areas involved in non-image forming visual system (dLGN, IGL, vLGN, OPN, SC, and opt) and the suprachiasmatic nucleus (SCN). Photomicrographs of each region were taken using a Leica light microscope and compared to MR images to validate regional identification.

### Statistical analysis

General activity data were analyzed using a mixed ANOVA with Greenhouse-Geisser adjustment for main effects of time (within subject) and age (between subjects) and their interaction on general activity measures. Where significant interactions between time and age were found, comparisons between age groups for each timepoint were analyzed using independent samples *t*-tests. For circadian rhythms analysis, independent samples *t*-tests were used to analyze onsets/offsets of activity, alpha, rho, precision, and cosinor variables. To compare manual and automated sleep scoring we used simple linear regressions with robust standard errors to adjust for heteroscedasticity. We also compared differences in bout length and number using independent samples *t*-tests. Masking was examined within the age groups independently, with repeated measure one-way ANOVAs and then, if significant, compared with paired samples *t*-tests. Histology of the retina was compared using an independent samples *t*-test. Finally, all volume differences were compared using independent samples *t*-tests. All analyses were performed with SPSS Statistic 23 software (IBM Corp., Armonk, NY) or STATA 16 software (College Station, TX) and significance for all tests was set at *p* < 0.05.

### Reporting summary

Further information on research design is available in the Nature Research Reporting Summary linked to this article.

## Supplementary information

Reporting Summary

Supplemental Video 1

Supplemental Material

## Data Availability

The data that support the findings of this study are available from the corresponding author upon request.

## References

[1] Bailey KJ, Maslov AY, Pruitt SC (2004). Accumulation of mutations and somatic selection in aging neural stem/progenitor cells. Aging Cell.

[2] Blackburn EH, Epel ES, Lin J (2015). Human telomere biology: a contributory and interactive factor in aging, disease risks, and protection. Science.

[3] Smith GL, Smith BD (2014). Radiation treatment in older patients: a framework for clinical decision making. J. Clin. Oncol..

[4] Parkes K (2002). Age, smoking, and negative affectivity as predictors of sleep patterns among shift workers in two environments. J. Occup. Health Psychol..

[5] Lai XY (2018). Sleep disturbance and related factors in patients with nasopharyngeal carcinoma and their family caregivers prior to the initiation of treatment. Sci. Rep..

[6] Dijk DJ, Duffy JF, Riel E, Shanahan TL, Czeisler CA (1999). Ageing and the circadian and homeostatic regulation of human sleep during forced desynchrony of rest, melatonin and temperature rhythms. J. Physiol..

[7] Schmidt C, Peigneux P, Cajochen C (2012). Age-related changes in sleep and circadian rhythms: impact on cognitive performance and underlying neuroanatomical networks. Front. Neurol..

[8] Van Cauter E, Leproult R, Plat L (2000). Age-related changes in slow wave sleep and REM sleep and relationship with growth hormone and cortisol levels in healthy men. Jama.

[9] Ingram DK, London ED, Reynolds MA (1982). Circadian rhythmicity and sleep: effects of aging in laboratory animals. Neurobiol. Aging.

[10] Hasan S, Dauvilliers Y, Mongrain V, Franken P, Tafti M (2012). Age-related changes in sleep in inbred mice are genotype dependent. Neurobiol. Aging.

[11] Wimmer ME (2013). Aging in mice reduces the ability to sustain sleep/wake states. PLoS ONE.

[12] Smagula SF (2016). Actigraphy and polysomnography measured sleep disturbances, inflammation, and mortality among older men. Psychosom. Med..

[13] Van Coevorden A (1991). Neuroendocrine rhythms and sleep in aging men. Am. J. Physiol. Endocrinol. Metab..

[14] Turek FW, Penev P, Zhang Y, Van Reeth O, Zee P (1995). Effects of age on the circadian system. Neurosci. Biobehav. Rev..

[15] Zee PC, Rosenberg RS, Turek FW (1992). Effects of aging on entrainment and rate of resynchronization of circadian locomotor activity. Am. J. Physiol. Regul. Integr. Comp. Physiol..

[16] Rappold I, Erkert HG (1994). Re‐entrainment, phase‐response and range of entrainment of circadian rhythms in Owl Monkeys (Aotus lemurinus g.) of different age. Biol. Rhythm Res..

[17] Sellix MT (2012). Aging differentially affects the re-entrainment response of central and peripheral circadian oscillators. J. Neurosci..

[18] Davidson AJ, Yamazaki S, Arble DM, Menaker M, Block GD (2008). Resetting of central and peripheral circadian oscillators in aged rats. Neurobiol. aging.

[19] Mohawk JA, Green CB, Takahashi JS (2012). Central and peripheral circadian clocks in mammals. Annu. Rev. Neurosci..

[20] Zhao J, Warman GR, Cheeseman JF (2019). The functional changes of the circadian system organization in aging. Ageing Res. Rev..

[21] Weinert H, Weinert D, Schurov I, Maywood ES, Hastings MH (2001). Impaired expression of the mPer2 circadian clock gene in the suprachiasmatic nuclei of aging mice. Chronobiol. Int..

[22] Wise PM, Cohen IR, Weiland NG, London ED (1988). Aging alters the circadian rhythm of glucose utilization in the suprachiasmatic nucleus. Proc. Natl Acad. Sci..

[23] Satinoff E (1993). Do the suprachiasmatic nuclei oscillate in old rats as they do in young ones?. Am. J. Physiol. Regul. Integr. Comp. Physiol..

[24] Aujard F, Herzog ED, Block GD (2001). Circadian rhythms in firing rate of individual suprachiasmatic nucleus neurons from adult and middle-aged mice. Neuroscience.

[25] Yamazaki S (2002). Effects of aging on central and peripheral mammalian clocks. Proc. Natl Acad. Sci..

[26] Ibuka N, Shin-ichi TI, Kawamura H (1977). Analysis of sleep-wakefulness rhythms in male rats after suprachiasmatic nucleus lesions and ocular enucleation. Brain Res..

[27] Göz D (2008). Targeted destruction of photosensitive retinal ganglion cells with a saporin conjugate alters the effects of light on mouse circadian rhythms. PLoS ONE.

[28] Güler AD (2008). Melanopsin cells are the principal conduits for rod–cone input to non-image-forming vision. Nature.

[29] Hatori M (2008). Inducible ablation of melanopsin-expressing retinal ganglion cells reveals their central role in non-image forming visual responses. PLoS ONE.

[30] Semo MA (2003). Melanopsin retinal ganglion cells and the maintenance of circadian and pupillary responses to light in aged rodless/coneless (rd/rd cl) mice. Eur. J. Neurosci..

[31] Nadal-Nicolás FM, Vidal-Sanz M, Agudo-Barriuso M (2018). The aging rat retina: from function to anatomy. Neurobiol. Aging.

[32] Pittendrigh CS, Daan S (1976). A functional analysis of circadian pacemakers in nocturnal rodents. J. Comp. Physiol..

[33] Daan S, Oklejewicz M (2003). The precision of circadian clocks: assessment and analysis in Syrian hamsters. Chronobiol. Int..

[34] Shuboni-Mulligan DD, Cavanaugh BL, Tonson A, Shapiro EM, Gall AJ (2019). Functional and anatomical variations in retinorecipient brain areas in Arvicanthis niloticus and Rattus norvegicus: implications for the circadian and masking systems. Chronobiol. Int..

[35] Armstrong TS (2017). Sleep-wake disturbance in patients with brain tumors. Neuro-Oncology.

[36] Dhruva A (2012). A longitudinal study of measures of objective and subjective sleep disturbance in patients with breast cancer before, during, and after radiation therapy. J. Pain Symptom Manag..

[37] Butt Z (2010). Age-associated differences in fatigue among patients with cancer. J. Pain Symptom Manag..

[38] Moscardo E, Rostello C (2010). An integrated system for video and telemetric electroencephalographic recording to measure behavioural and physiological parameters. J. Pharmacol. Toxicol. Methods.

[39] Ferreira A (2006). Spontaneous appetence for wheel-running: a model of dependency on physical activity in rat. Eur. Psychiatry.

[40] Brené S (2007). Running is rewarding and antidepressive. Physiol. Behav..

[41] Nakamura TJ (2011). Age-related decline in circadian output. J. Neurosci..

[42] Valentinuzzi VS, Scarbrough K, Takahashi JS, Turek FW (1997). Effects of aging on the circadian rhythm of wheel-running activity in C57BL/6 mice. Am. J. Physiol. Regul. Integr. Comp. Physiol..

[43] Aschoff J (1999). Masking and parametric effects of high-frequency light-dark cycles. Jpn J. Physiol..

[44] Redlin U (2001). Neural basis and biological function of masking by light in mammals: suppression of melatonin and locomotor activity. Chronobiol. Int..

[45] Herbst K (2012). Intrinsically photosensitive retinal ganglion cell function in relation to age: a pupillometric study in humans with special reference to the age-related optic properties of the lens. BMC Ophthalmol..

[46] Adhikari P, Pearson CA, Anderson AM, Zele AJ, Feigl B (2015). Effect of age and refractive error on the melanopsin mediated post-illumination pupil response (PIPR). Sci. Rep..

[47] Banks G (2015). Genetic background influences age-related decline in visual and nonvisual retinal responses, circadian rhythms, and sleep. Neurobiol. Aging.

[48] Seibert TM (2017). Radiation dose-dependent hippocampal atrophy detected with longitudinal volumetric magnetic resonance imaging. Int. J. Radiat. Oncol. Biol. Phys..

[49] Brown PD (2020). Hippocampal avoidance during whole-brain radiotherapy plus memantine for patients with brain metastases: phase III trial NRG Oncology CC001. J. Clin. Oncol..

[50] Ystad MA (2009). Hippocampal volumes are important predictors for memory function in elderly women. BMC Med. Imaging.

[51] Terribilli D (2011). Age-related gray matter volume changes in the brain during non-elderly adulthood. Neurobiol. Aging.

[52] Nobis L (2019). Hippocampal volume across age: nomograms derived from over 19,700 people in UK Biobank. NeuroImage.

[53] Stephan H, Frahm H, Baron G (1984). Comparison of brain structure volumes in insectivora and primates. IV. Non-cortical visual structures. J. Hirnforsch..

[54] Edelstein K, Mrosovsky N (2001). Behavioral responses to light in mice with dorsal lateral geniculate lesions. Brain Res..

[55] Langel J, Ikeno T, Yan L, Nunez AA, Smale L (2018). Distributions of GABAergic and glutamatergic neurons in the brains of a diurnal and nocturnal rodent. Brain Res..

[56] Zhang Z (2019). Superior colliculus GABAergic neurons are essential for acute dark induction of wakefulness in mice. Curr. Biol..

[57] Gall AJ, Goodwin AM, Khacherian OS, Teal LB (2019). (2019). Superior colliculus lesions lead to disrupted responses to light in diurnal grass rats (Arvicanthis niloticus). J. Biol. Rhythms.

[58] Kolker DE, Vitaterna MH, Fruechte EM, Takahashi JS, Turek FW (2004). Effects of age on circadian rhythms are similar in wild-type and heterozygous clock mutant mice. Neurobiol. Aging.

[59] Lupi D, Foster RG (2012). Impact of age and retinal degeneration on the light input to circadian brain structures. Neurobiol. Aging.

[60] Goldby F (1957). A note on transneuronal atrophy in the human lateral geniculate body. J. Neurol. Neurosurg. Psychiatry.

[61] Kelly KR, McKetton L, Schneider KA, Gallie BL, Steeves JK (2014). Altered anterior visual system development following early monocular enucleation. NeuroImage.

[62] Yang XL (2018). Age-related changes in eye, brain and visuomotor behavior in the DBA/2J mouse model of chronic glaucoma. Sci. Rep..

[63] Cavallotti D, Cavallotti C, Pescosolido N, Iannetti GD, Pacella E (2001). A morphometric study of age changes in the rat optic nerve. Ophthalmologica.

[64] Sandell JH, Peters A (2002). Effects of age on the glial cells in the rhesus monkey optic nerve. J. Comp. Neurol..

[65] Shuboni DD, Cramm S, Yan L, Nunez AA, Smale L (2012). Acute behavioral responses to light and darkness in nocturnal Mus musculus and diurnal Arvicanthis niloticus. J. Biol. Rhythms.

[66] Fogo GM, Shuboni-Mulligan DD, Gall AJ (2019). Melanopsin-containing ipRGCs are resistant to excitotoxic injury and maintain functional non-image forming behaviors after insult in a diurnal rodent model. Neuroscience.

[67] Gall AJ, Shuboni DD, Yan L, Nunez AA, Smale L (2016). Suprachiasmatic nucleus and subparaventricular zone lesions disrupt circadian rhythmicity but not light-induced masking behavior in Nile grass rats. J. Biol. Rhythms.

[68] Young D (2019). Dose response curve for radiation-induced hypersomnolence (RIH) in a mouse model of cranial radiation: behavioral analysis of sleep and activity. Neuro-Oncology.

[69] Schindelin J (2012). Fiji: an open-source platform for biological-image analysis. Nat. Methods.

[70] Fedorov A (2012). 3D slicer as an image computing platform for the quantitative imaging network. Magn. Reson. Imaging.

