# Supplementary material for: Impact of age on the circadian visual system and the sleep-wake cycle in mus musculus
Source: NPJ Aging Mech Dis. 2021 May 4;7:10. doi: 10.1038/s41514-021-00063-w (PMC8096965; doi:10.1038/s41514-021-00063-w)
Supplement: Supplementary file 1 — Reporting Summary [file 41514_2021_63_MOESM1_ESM.pdf]

nature research

Corresponding author(s): Dr Dorela Shuboni-Mulligan

Last updated by author(s): Nov 11, 2020

Reporting Summary

Nature Research wishes to improve the reproducibility of the work that we publish. This form provides structure for consistency and transparency in reporting. For further information on Nature Research policies, see our Editorial Policies and the Editorial Policy Checklist.

Statistics

For all statistical analyses, confirm that the following items are present in the figure legend, table legend, main text, or Methods section.

n/a	Confirmed	
☐	☒	The exact sample size (*n*) for each experimental group/condition, given as a discrete number and unit of measurement
☐	☒	A statement on whether measurements were taken from distinct samples or whether the same sample was measured repeatedly
☐	☒	The statistical test(s) used AND whether they are one- or two-sided<br>*Only common tests should be described solely by name; describe more complex techniques in the Methods section.*
☒	☐	A description of all covariates tested
☐	☒	A description of any assumptions or corrections, such as tests of normality and adjustment for multiple comparisons
☐	☒	A full description of the statistical parameters including central tendency (e.g. means) or other basic estimates (e.g. regression coefficient) AND variation (e.g. standard deviation) or associated estimates of uncertainty (e.g. confidence intervals)
☐	☒	For null hypothesis testing, the test statistic (e.g. *F*, *t*, *r*) with confidence intervals, effect sizes, degrees of freedom and *P* value noted<br>*Give P values as exact values whenever suitable.*
☒	☐	For Bayesian analysis, information on the choice of priors and Markov chain Monte Carlo settings
☒	☐	For hierarchical and complex designs, identification of the appropriate level for tests and full reporting of outcomes
☒	☐	Estimates of effect sizes (e.g. Cohen's *d*, Pearson's *r*), indicating how they were calculated

Our web collection on statistics for biologists contains articles on many of the points above.

Software and code

Policy information about availability of computer code

Data collection	Ethovision XT 14 with Mouse behavior Module and ParaVision 5
Data analysis	IBM SPSS, FIJI, Cosinor Software (Roberto Refinetti, circadian.org), and 3D Slicer 4.8

For manuscripts utilizing custom algorithms or software that are central to the research but not yet described in published literature, software must be made available to editors and reviewers. We strongly encourage code deposition in a community repository (e.g. GitHub). See the Nature Research guidelines for submitting code & software for further information.

Data

Policy information about availability of data

All manuscripts must include a data availability statement. This statement should provide the following information, where applicable:

- Accession codes, unique identifiers, or web links for publicly available datasets
- A list of figures that have associated raw data
- A description of any restrictions on data availability

The data that support the findings of this study are available from the corresponding author upon request.

Field-specific reporting

Life sciences study design

All studies must disclose on these points even when the disclosure is negative.

Sample size	Sample sizes were determined by the authors experience with circadian rhythms, needing a minimum of 8 mice per group.
Data exclusions	No available data was excluded from the experiments.
Replication	We are currently using the video scoring system in subsequent experiments and find similar results in young mice. The results of the study find similar but more detailed findings as those reported in the literature.
Randomization	The experiment examined age and could therefore not be randomly assigned.
Blinding	Scorers of behavior and brain volume size were blinded to the groups during the process.

Reporting for specific materials, systems and methods

We require information from authors about some types of materials, experimental systems and methods used in many studies. Here, indicate whether each material, system or method listed is relevant to your study. If you are not sure if a list item applies to your research, read the appropriate section before selecting a response.

Materials & experimental systems

n/a	Involved in the study	
x		Antibodies
x		Eukaryotic cell lines
x		Palaeontology and archaeology
	x	Animals and other organisms
x		Human research participants
x		Clinical data
x		Dual use research of concern

Methods

n/a	Involved in the study	
x		ChIP-seq
x		Flow cytometry
	x	MRI-based neuroimaging

Animals and other organisms

Policy information about studies involving animals; ARRIVE guidelines recommended for reporting animal research

Laboratory animals	Male C57BL/6 Mice at 6 weeks and 18 months old
Wild animals	n/a
Field-collected samples	n/a
Ethics oversight	IACUC at the NCI, NIH Bethesda MD

Note that full information on the approval of the study protocol must also be provided in the manuscript.

Magnetic resonance imaging

Experimental design

Design type	Ex vivo Structural MRI
Design specifications	Mice were perfused with Gad doped Para-formaldehyde and placed in Flourinert and scanned with a 14.1T Bruker Microimaging.
Behavioral performance measures	n/a

Acquisition

Imaging type(s)	Structural
Field strength	14.1
Sequence & imaging parameters	FLASH sequence, TE=5ms, TR=50ms, FOV=1.6x0.8x0.8cm, Resolution=32x32x32um, Averages=10
Area of acquisition	Whole brain

Diffusion MRI ☐ Used ☒ Not used

Preprocessing

Preprocessing software	FIJI and 3D Slicer 4.8
Normalization	n/a
Normalization template	n/a
Noise and artifact removal	n/a
Volume censoring	n/a

Statistical modeling & inference

Model type and settings	n/a
Effect(s) tested	n/a

Specify type of analysis: ☐ Whole brain ☐ ROI-based ☒ Both

Anatomical location(s): A neuro-anatomical expert identified structures and defined them manually.

Statistic type for inference (See Eklund et al. 2016)	n/a
Correction	n/a

Models & analysis

n/a	Involved in the study
☒	☐ Functional and/or effective connectivity
☒	☐ Graph analysis
☒	☐ Multivariate modeling or predictive analysis
