# Supplementary material for: Impact of age on the circadian visual system and the sleep-wake cycle in mus musculus
Source: NPJ Aging Mech Dis. 2021 May 4;7:10. doi: 10.1038/s41514-021-00063-w (PMC8096965; doi:10.1038/s41514-021-00063-w)
Supplement: Supplementary file 3 — Supplemental Material [file 41514_2021_63_MOESM3_ESM.docx]

Supplemental Figure:


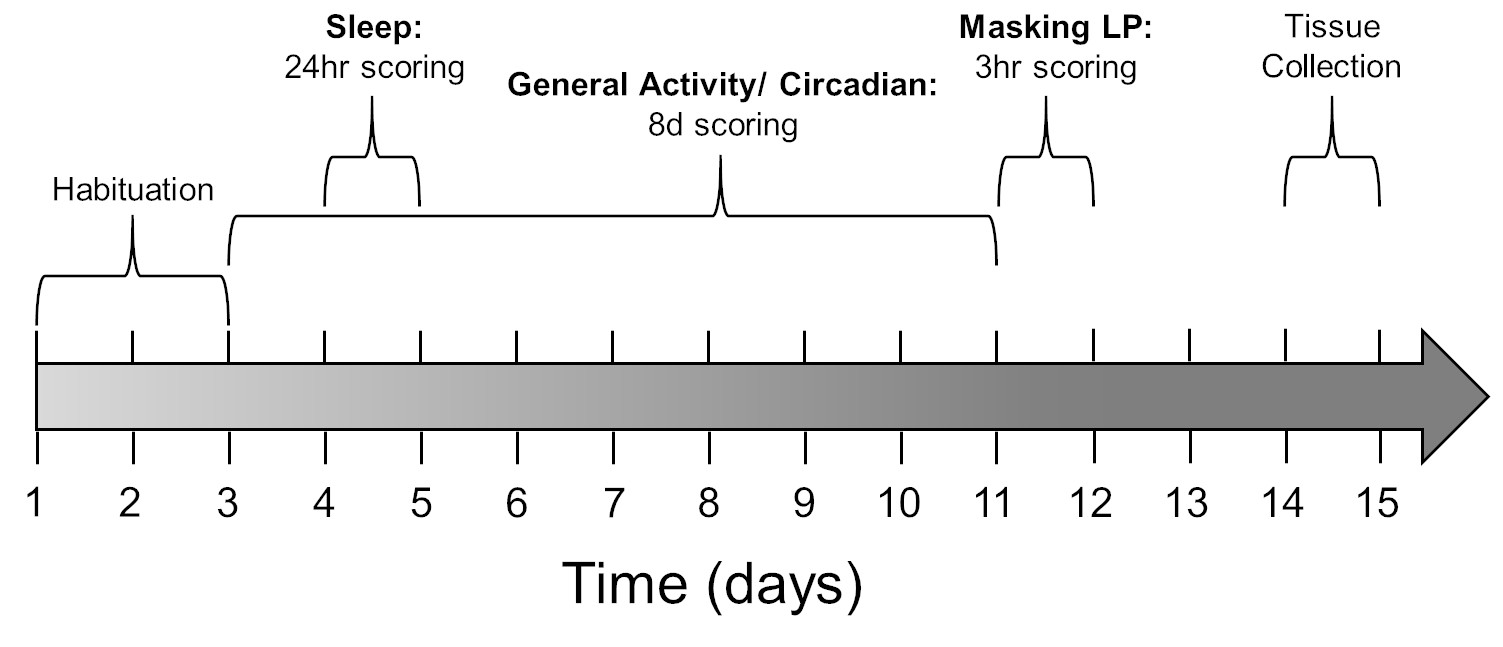


Supplemental Figure 1: The experimental timeline of behavioral analysis and tissue collection. Animals were allowed to habituate for 3 days, our laboratory observed heightened activity levels over the first 2-3 days of introduction to the novel video cage setting. General activity analysis was averaged across 8 days of monitoring, including distance travelled, movement and velocity and were quantified at the hour and day/night levels. Sleep analysis between video and automated program was compared for 24 hours on day 4. Masking analysis was conducted on day 11, with 1 hour of light pulse at zeitgeber time 14 (2 hours after lights off). Tissue was collected after 3 days.


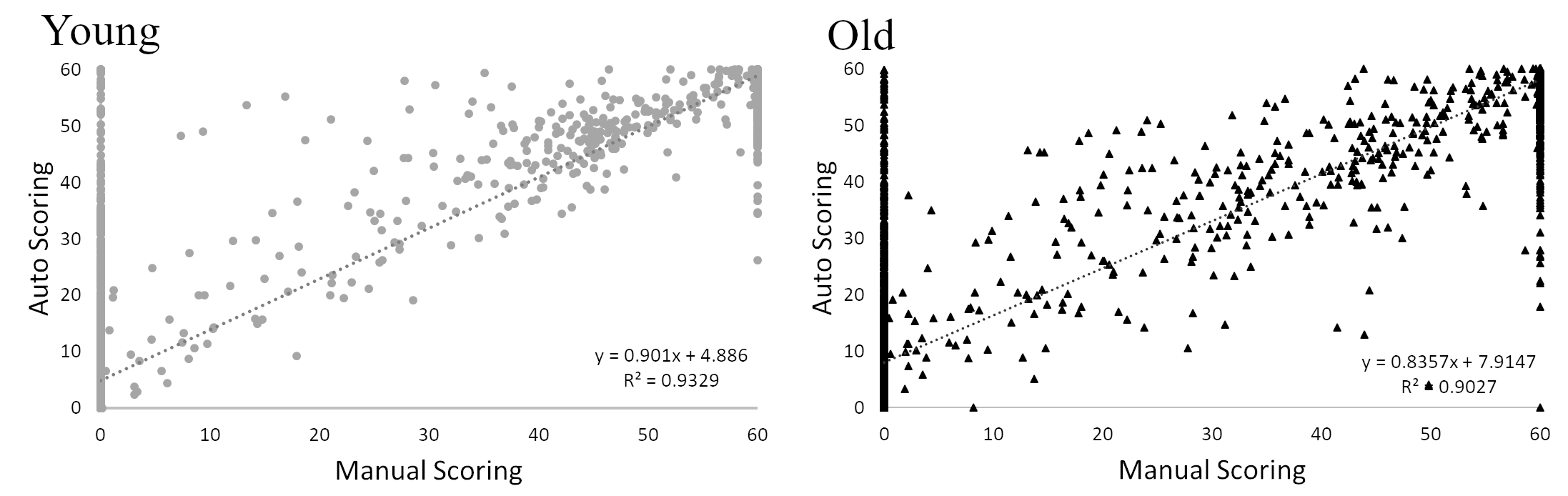


Supplemental Figure 2: Comparisons between automated and manual scoring at the one-minute level for young (left) and old (right) mice.

Supplemental Video: A compilation of behaviors using the automated video recording systems including Sleep-like behavior, Sleep Movement, Rest, Activity, Grooming, Nesting, Eating and Drinking.
